# Supplementary material for: Modeling hepatitis D virus kinetics during bulevirtide monotherapy: challenges and solutions
Source: ArXiv. 2026 Jun 4:arXiv:2505.12286v2. Preprint. [Version 2] (PMC13252502)
Supplement: Supplement 1 [file NIHPP2505.12286v2-supplement-1.pdf]

Supplementary Materials (SM)

Supplementary Material Figures

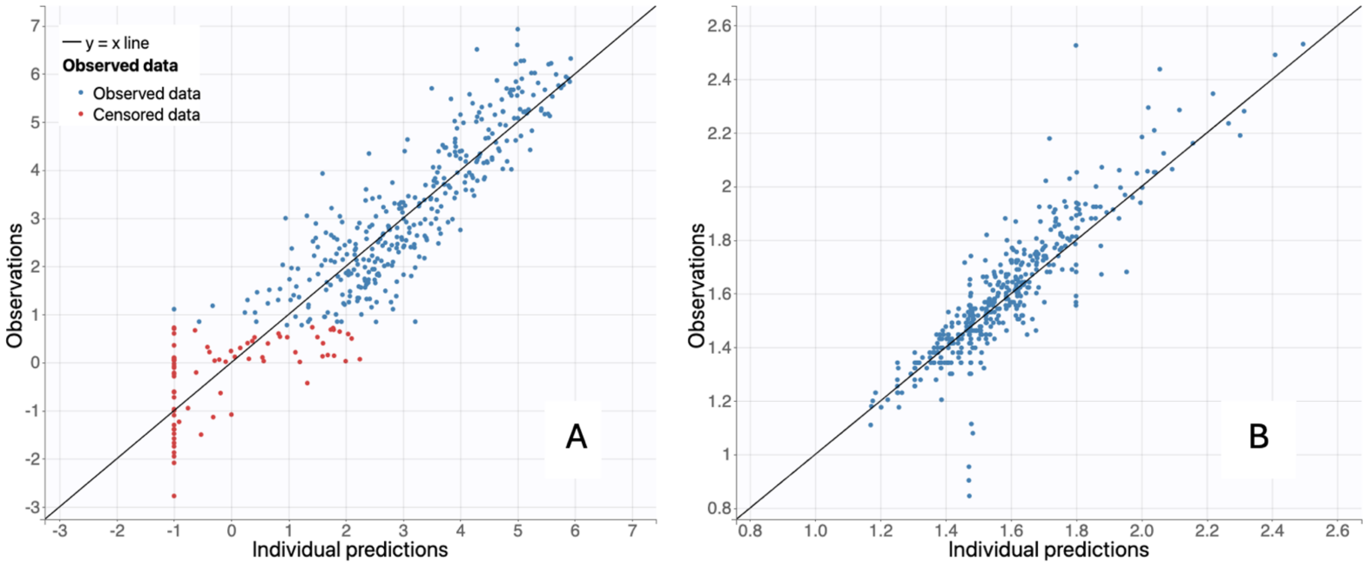

Supplementary Fig. 1: Plot of observations vs. individual predictions for **A** (HDV) and **B** (ALT), illustrating the relationship between observation and predictions and helping to identify potential issues with the structural model.

In **Supplementary Fig. 1A**, for HDV, the measured data points range from 4.5 to 5.5 and are not symmetrically spread around the diagonal, suggesting that the model does not accurately capture the data. Using the interactive feature in Monolix on the predictions vs. observations plots, we observed that this asymetry is because of non-monophasic declines, which were not fit well. In **Supplementary Fig. 1B**, we overall observe good agreement between the model and ALT data except in one isolated case.

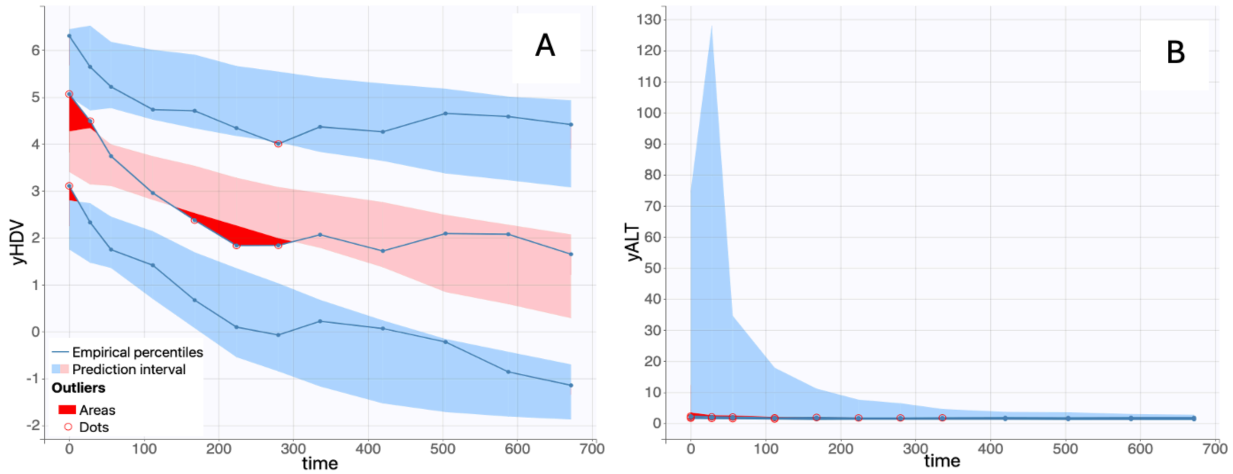

Supplementary Fig. 2: The ability of model (Eq. 1) to explain the data, as assesed by the visual predictive check (VPC).

The VPC is based on multiple simulations with the model and the design structure of the observed data, grouped in bins over successive time intervals with optimized binning criteria. Both Figures, **Supplementary Fig. 2A** and **2B** show that the model has good predictive power, however, its accuracy is limited by the presence of minor outliers.

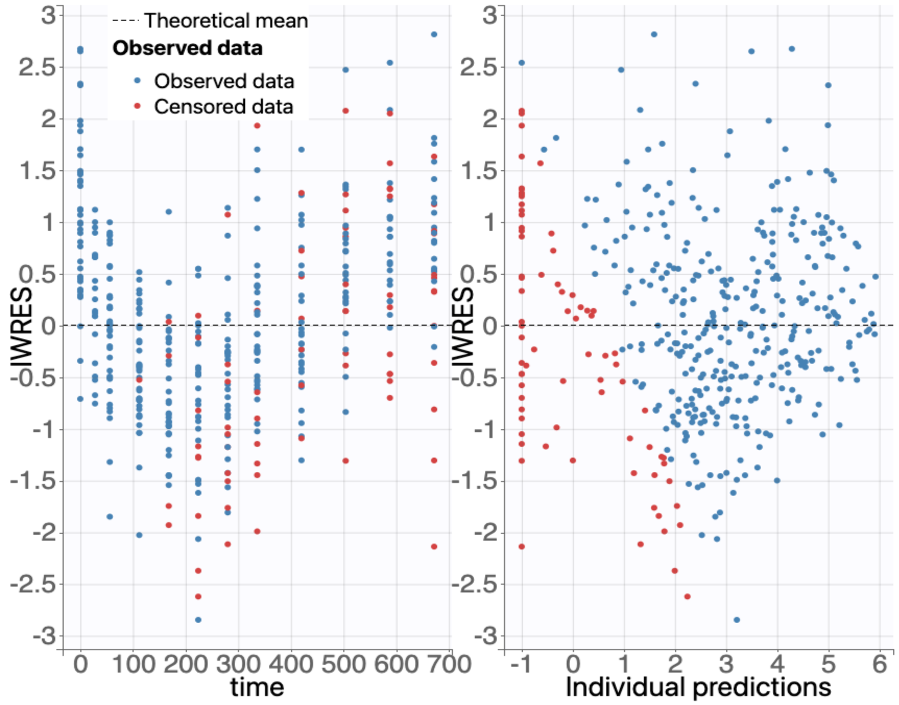

Supplementary Fig. 3: Diagnostic plots for model fit assessment for HDV. **(left)** Individual weighted residuals (IWRES) vs. time, denotes the disribution of individual weighted residuals over time. Ideally, the model should be randomly scattered around zero indicating an appropriate model fit with no time-related biases. **(right)** IWRES vs individual predictions, illustrating residuals in relation to individual predicted value. Ideally, residuals should be evenly distributed around zero without trends, suggesting accurate predictions across the full data set.

In **Supplementary Fig. 3**, the IWRES vs. time plot shows residuals scatterred around zero, although some clustering occurs, suggesting potential time-related model misspecifications. For the IWRES vs. individual predictions plot, the residuals are not symmetrical, indicating that the model may not fully capture variabiliry across the prediction range.

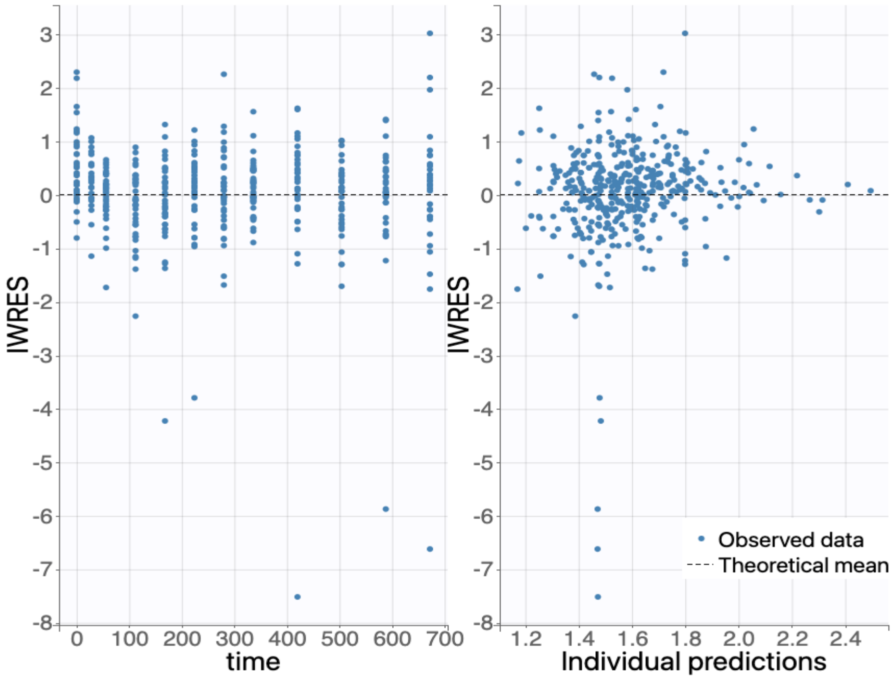

Supplementary Fig. 4: Diagnostic plots for model fit assessment for ALT. **(left)** IWRES vs. time, shows the disribution of individual weighted residuals over time; a random scatter around zero indicates an appropriate model fit without time-related biases. **(right)** IWRES vs. individual predictions, capturing residuals compared to individual predicted values. Ideally, residuals should be evenly distributed around zero without any patterns, suggesting accurate predictions across the range of the data.

In **Supplementary Fig. 4**, we see that in the IWRES vs. time plot, most residuals are clustered around zero, meaning that the model can explain the variability well, though a few outliers indicate potential deviations. In the IWRES vs Individual predictions plot, residuals cluster tightly around zero for most of the range, while some extreme residuals at lower and higher values indicate possible minor inaccuracie
